# Intra-mesenteric steroids for steroid-refractory graft-versus-host disease in pediatric patients: A safe option

**DOI:** 10.7705/biomedica.7394

**Published:** 2024-12-23

**Authors:** Ana M. Aristizábal, Lina P. Montaña, Jaiber Gutiérrez, Diego Medina, Alexis A. Franco, Eliana Manzi, Ángela Devia Zapata, Walter Mosquera

**Affiliations:** 1 Servicio de Cardiología Pediátrica, Departamento Materno-Infantil, Fundación Valle del Lili, Cali, Colombia Fundación Valle del Lili Cali Colombia; 2 Facultad de Ciencias de la Salud, Universidad Icesi, Cali, Colombia Universidad Icesi Universidad Icesi Cali Colombia; 3 Unidad de Trasplante de Médula Ósea, Servicio de Hematoncología Pediátrica, Departamento Materno-Infantil, Fundación Valle del Lili, Cali, Colombia Fundación Valle del Lili Cali Colombia; 4 Centro de Investigaciones Clínicas, Fundación Valle del Lili, Cali, Colombia Fundación Valle del Lili Cali Colombia

**Keywords:** graft-versus-host disease, steroids, injections, intra-arterial, bone marrow transplantation, pediatrics, enfermedad injerto contra huésped, esteroides, inyecciones intraarteriales, trasplante de médula ósea, pediatría

## Abstract

**Introduction.:**

Graft-versus-host disease is a serious complication after hematopoietic stem cell transplantation and is a major cause of death post-transplantation. Approximately 50% of acute graft-versus-host disease patients do not respond to systemic steroids and their prognosis is poor regardless of the treatment. This study describes our experience with pediatric patients diagnosed with steroid-refractory graft-versus-host disease who received intra-mesenteric steroid treatment.

**Objective.:**

To determine the outcomes of intra-mesenteric steroid use in the management of pediatric patients diagnosed with refractory graft-versus-host disease.

**Materials and methods.:**

The study included patients under 18 years old with allogeneic hematopoietic stem cell transplantation who underwent intra-mesenteric steroid injection for resistant gastrointestinal graft-versus-host disease between January, 2016, and December, 2021. Methylprednisolone was administered via intra-arterial injection through the celiac trunk and the superior and inferior mesenteric arteries.

**Results.:**

We collected data on 21 patients: nine (90%) responded with a subjective decrease in fecal output and a reduction in bilirubin and transaminases. Seven patients required a second intra-mesenteric injection and presented a complete response in 85% of the cases. Only one patient experienced local complications after the procedure. Twelve patients (57%) died with one death due to acute graft-versus-host disease.

**Conclusion.:**

Reports in the adult population have shown an approximately 50% response rate with few complications, making it a second-line management standard. As far as we know, this is the largest pediatric cohort reported in Latin America. Our findings suggest that intra-mesenteric steroid administration for managing hepatic and gastrointestinal graft- versus-host disease may be considered an early adjuvant treatment in patients with steroid- refractory graft-versus-host disease.

## Resumen

**Introducción.:**

La enfermedad de injerto contra huésped es una complicación grave tras un trasplante de células madre hematopoyéticas y es una de las principales causas de muerte después del trasplante. Alrededor del 50 % de los pacientes con enfermedad aguda de injerto contra huésped no mejora con los esteroides sistémicos y su pronóstico es desfavorable, independientemente del tratamiento.

En este estudio, se describe la experiencia de la Fundación Valle del Lili con pacientes menores de 18 años con diagnóstico de enfermedad de injerto contra huésped resistente a esteroides que recibieron tratamiento con esteroides intramesentéricos.

**Objetivo.:**

Determinar los resultados del uso de esteroides intramesentéricos en el tratamiento de pacientes pediátricos con diagnóstico de enfermedad de injerto contra huésped resistente a esteroides.

**Materiales y métodos.:**

Se incluyeron pacientes menores de 18 años con trasplante alogénico de progenitores hematopoyéticos, sometidos a inyección intramesentérica de esteroides para enfermedad gastrointestinal de injerto contra huésped resistente, entre enero del 2016 y diciembre del 2021. La metilprednisolona fue administrada por inyección intraarterial en el tronco celiaco y las arterias mesentéricas superior e inferior.

**Resultados.:**

Se recolectaron datos de 21 pacientes, de los cuales 19 (90 %) presentaron mejoría con disminución subjetiva de la producción fecal, y reducción de la bilirrubina y las transaminasas. Siete pacientes requirieron una segunda inyección intramesentérica y presentaron mejoría completa en el 85 % de los casos. Solo un paciente tuvo complicaciones locales tras el procedimiento y 12 pacientes (57 %) fallecieron debido al desarrollo de enfermedad gastrointestinal aguda de injerto contra huésped.

**Conclusión.:**

Los informes sobre población adulta han reportado una tasa de mejoría del 50 %, aproximadamente, con pocas complicaciones, lo que convierte a la inyección intramesentérica de esteroides en un estándar terapéutico de segunda línea.

Hasta donde se sabe, esta es la cohorte pediátrica más grande reportada en Latinoamérica. Los hallazgos de este estudio sugieren que la administración intramesentérica de esteroides para el manejo de enfermedad hepática y gastrointestinal de injerto contra huésped puede considerarse un tratamiento adyuvante temprano en pacientes con este tipo de enfermedad resistente a los esteroides sistémicos.

Graft-versus-host disease affects 30 to 80% of hematopoietic stem cell transplantation recipients ^1^, and it is the principal cause of non-relapse mortality after this type of transplantation ^2^^,^^3^. This pathology produces a severe inflammatory process, affecting the skin, the gastrointestinal system, and the liver ^4^.

The standard treatment is systemic steroids; however, approximately in 50% of patients the disease will be steroid-refractory ^5^^,^^6^. Different second- line systemic therapies have been used ^7^. All these agents increase immunosuppression and predispose the patient to infections and secondary neoplasms ^8^. The prognosis of steroid-resistant or dependent graft-versus- host disease tends to be poor, independently of the choice of systemic treatment ^9^^,^^10^.

There is a critical need to establish a standard treatment for severe graft- versus-host disease and to continue advancing in prevention strategies ^11^^,^^12^. Various groups have demonstrated that infusing steroids directly into the artery supplying the organ affected by the disease (such as the liver or intestine) is safe and can induce its remission ^2^^,^^13^^-^^15^. The localized injection of steroids aims to deliver a higher dose of the drug to the target organ, achieving low systemic levels and avoiding related unwanted effects ^16^^-^^19^.

Our institution is a reference center in the country for performing hematopoietic stem cell transplantation in pediatric patients. Since 2016, we have used intra-mesenteric steroid injections for pediatric patients post- transplantaion with gastrointestinal graft-versus-host disease, administered by pediatric interventional cardiologists. Currently, in patients diagnosed with moderate-to-severe disease who do not respond to the initial management, we use early intra-mesenteric steroid injection.

Limited data exists on using steroids to manage refractory graft-versus- host disease in children ^20^. This study aimed to describe the clinical outcomes and our experience with 21 pediatric patients diagnosed with steroid-refractory graft-versus-host disease who received intra-mesenteric steroid treatment. To our knowledge, this is the largest pediatric cohort reported in Latin America.

## Materials and methods

### Patients

This is a retrospective observational case series that included patients under 18 years who received allogenic hematopoietic stem cell transplantation for any indication and required intra-mesenteric steroid injection for therapy-resistant moderate to severe gastrointestinal graft- versus-host disease between January 2016 and December 2021.

Steroid-refractory was defined as the progression of the disease after three days, lack of response after seven days, or incomplete response after 14 days of initiating the standard treatment. Steroid dependence was defined as a response that reappears when the steroid dose is decreased ^21^.

Graft-versus-host disease prophylaxis was assigned according to the donor type: cyclophosphamide for haploidentical donors ^21^; and calcineurin inhibitors, and methotrexate or mycophenolate, for identical donors ^22^.

Methylprednisolone was administered via intra-mesenteric injection into the celiac trunk and the superior and inferior mesenteric arteries, at 1 mg/kg (up to a maximum of 60 mg per artery) diluted in 10 ml of 0.9% saline solution.

Thrombocytopenia did not contraindicate the procedure. Patients with platelet counts below 50,000 cells/μΙ were transfused on the day of the procedure to achieve a safe value before the intervention.

### Technique used

Using the Seldinger technique, the femoral artery was catheterized using 4- or 5-Fr introducers, depending on the patient’s age and weight. A 4- or 5-Fr pigtail catheter was then advanced to perform aortic angiography and locate the celiac trunk, and the superior and inferior mesenteric artery.

**Figure 1:**
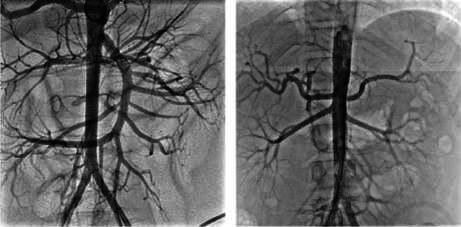
Intra-arterial steroid injection. Proof of correct catheter placement during intra-arterial steroid injection: celiac trunk (left), inferior mesenteric artery (right).

Once these arteries were located, a 4- or 5-Fr Cobra catheter was used to catheterize individually the celiac trunk and the superior mesenteric artery. If catheterization of the inferior mesenteric artery was not feasible with the cobra catheter, a Sigmon 1 catheter was used as an alternative. Then, methylprednisolone was injected into each artery for three minutes per injection ^4^^,^^23^ (figure 1).

After the procedure, the patients continued receiving the front-line management established for graft-versus-host disease. Patients with partial response received a second dose two weeks after the first application. Patients were on bed rest with restricted limb flexion for two to six hours after the injection. Associated complications were addressed after the intervention (hematoma, arterial bleeding, dissection or thrombosis, arterial spasm, and cellulitis).

### Variables

Clinical and demographic characteristics of the patients were collected, along with outcomes associated with the intra-mesenteric steroid injection procedure and with the post-treatment of gastrointestinal graft-versus-host disease, overall survival and mortality related to relapse 24 months after hematopoietic stem cell transplantation.

Response time was defined as the period between the day of the intramesenteric steroid treatment and the observed response in the next two weeks. Each affected organ was evaluated according to the following criteria:

- Hepatic: A decrease of 25% in serum bilirubin levels from their baselines was called “partial response”, and reaching normal bilirubin levels was denominated “complete response”.
- Gastrointestinal: A subjective improvement of diarrhea with reduced volume, frequency, and abdominal pain or the disappearance of bleeding was denominated “partial response”. The resolution of diarrhea and all other gastrointestinal symptoms was denominated “complete response”.

### Statistical analysis

Data distribution was assessed using the Shapiro-Wilk test, with a p value ≤ 0.05 considered significant. The null hypothesis was assumed if the data followed a normal distribution, while the alternative hypothesis was accepted if the data did not follow a normal distribution. When the variable followed a normal distribution, it was summarized using the mean as a central tendency measure and the standard deviation as a dispersion measure. Variables without a normal distribution were summarized using the median as a central tendency measure and the interquartile range as a dispersion measure. Univariate analysis of qualitative variables was summarized as percentages.

Furthermore, the bivariate analysis was used to explore possible associations, relations, or dependence among the exposure and outcome variables (improvement of the gastrointestinal graft-versus-host disease).

When the variables were nominal dichotomous, relative risk (RR) or odds ratio (OR) were used to measure association with their respective 95% confidence intervals. Statistical tests were applied to confirm a statistically significant association depending on the variables.

Event-free survival [death, overall survival (OS), graft failure, and incidence of moderate-to-severe gastrointestinal graft-versus-host disease) was assessed using the Kaplan-Meier method.

We used the log-rank test to compare transplant and diagnosis types. Finally, exploratory analysis of the entire database was conducted using the Stata™, version 12 statistical package.

### Ethics approval

The Institutional Review Board approved the study under approval number 1823, in accordance with the Declaration of Helsinki.

## Results

During the study, 21 pediatric patients with hematopoietic stem cell transplantation and a diagnosis of steroid-refractory graft-versus-host disease were referred for an intra-mesenteric steroid injection. The mean age at the time of transplantation was 10 ± 5 years. Of these transplantations, 90.5% were haploidentical, with peripheral blood being the primary source for transplantation (62%). Most patients had a malignant disease as a primary diagnosis (57%) (table 1).

**Table 1:** Clinical and demographic characteristics (n = 21)

Variable		Value n (%)
Age (years)		
	Mean	10 ± 5
	Range (min-max)	1-16
Sex (male)		14 (67)
Type of transplant		
	Matched sibling donors	2 (9.5)
	Haploidentical	19 (90.5)
Transplant source		
	Bone marrow	7 (33)
	Peripheral blood	13 (62)
	Bone marrow + peripheral blood	1 (5)
Diagnosis		
	Malignant	12 (57)
Conditioning, n (%)		
	Myeloablative	12 (57)
	Non-myeloablative	9 (43)

Of the 21 patients, 38% required blood products before the procedure. Nineteen (90%) patients had some degree of response to the steroid injection, including a subjective decrease in fecal output and reductions in bilirubin and transaminases within the two weeks following the application (table 2).

**Table 2:** Characteristics and complications of intramesenteric steroid injection (n = 21)

Variable		Value n (%)
Intra-mesenteric steroid injection #1		21
Initial response to the procedure		
	Complete	19 (90)
	Partial	2 (10)
Subjective decrease in diarrhea		18 (86)
Intramesenteric steroid injection #2		7
Response to the procedure		
	Complete	6 (86)
	Partial	1 (14)

Seven patients required a second intra-mesenteric steroid injection several weeks after the first application due to the recurrence of symptoms, with complete response in 85% of these cases (table 2).

**Figure 2:**
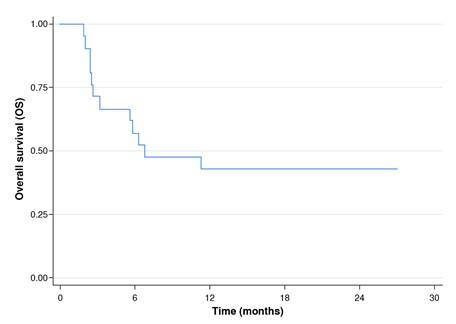
Overall survival in pediatric hematopoietic stem cell transplantation receptors subjected to intra-mesenteric steroid injection to treat graft-versus-host disease

Regarding complications related to hematopoietic stem cell transplantation, three patients experienced associated cytopenia. The overall survival rate was 67% at 100 days and 43% at 24 months (figure 2). Two of the patients were diagnosed with graft-versus-host disease in their second transplant. Out of all the patients, 12 (57%) died. Among the causes of death, seven cases were associated with infections; one was due to hemorrhagic cystitis, one was related to disease progression, and one was secondary to graft-versus-host disease (table 3).

**Table 3:** Complications related with hematopoietic stem cell transplantation (n = 21)

Variable		Value n (%)
Infection		
	Viral	17 (81)
	Bacterial	15 (71)
	Fungal	6 (28)
	Parasitic	3 (14)
Veno-occlusive hepatic disease		4 (19)
Mucositis		12 (57)
	Slight	3
	Moderate	5
	Severe	4
Hemorrhagic cystitis		9 (43)
Cytopenia		3 (14)
Death		12 (57)

## Discussion

Mortality rates up to 90% have been reported in patients with graft- versus-host disease refractory to steroids despite using different therapeutic strategies, including etanercept, infliximab, alemtuzumab, thymoglobulin, high doses of steroids, and others ^24^. However, several of these medications are not broadly available and have regulatory limitations for pediatric use. High mortality is widely associated with secondary infections ^25^; therefore, no consensus has been established for a standard second-line treatment ^9^^,^^10^.

Intra-mesenteric steroid injection has proven effective in patients diagnosed with severe ulcerative colitis ^26^. Some authors have extrapolated their use in patients with moderate-to-severe graft-versus-host disease, given its similar pathophysiological characteristics ^2^.

Reports in adult populations with hematopoietic stem cell transplantation and intra-mesenteric steroid injection show an approximately 50% response rate, with a low percentage of complications; thus, some institutions have adopted this therapy as a standard second-line management ^4^^,^^14^. Multiple reports demonstrate its safety and efficacy ^2^^,^^13^^,^^19^; however, there are few cases in pediatric patients ^4^^,^^20^.

In this case series study, we observed similar outcomes in pediatric patients at a high-complexity institution with low rates of complications. The survival rates of patients in this study were comparable to those reported for this intra-mesenteric injection therapy ^2^^,^^4^. Additionally, with only one patient experiencing local complications, the rate of such complications was low and consistent with findings from other studies.

In our patients, and as described globally, there was a significant decrease in overall survival percentage within the first 100 days after transplantation. However, there were no treatment-related deaths.

According to our results, all patients reported subjective improvement in symptoms, including a reduced frequency of bowel movements and a decreasing trend in direct bilirubin and transaminases. These findings are consistent with those reported in the literature, which suggests that intramesenteric steroid injection may also improve hepatic graft-versus-host disease ^2^^,^^9^.

Several limitations exist in our study, including its non-randomized design, its nature as a case series, and the fact that it was conducted in a single center.

Based on our findings, we consider that intra-mesenteric steroid administration for the management of hepatic or gastrointestinal graft-versus- host disease is a feasible option and can be considered as an early adjuvant to the initial treatment in patients with moderate-to-severe refractory graft- versus-host disease. Recently, promising results have been reported with the ruxolitinib use in some patients; however, its availability is still limited ^27^.

Further studies are necessary in the region to gather more evidence and develop protocols for managing refractory graft-versus-host disease in pediatric patients.
